# Changing Trends of Adverse Pregnancy Outcomes With Maternal Pre-pregnancy Body Mass Index: A Join-Point Analysis

**DOI:** 10.3389/fmed.2022.872490

**Published:** 2022-06-03

**Authors:** Rong Hu, Huifen Yin, Xiaotian Li

**Affiliations:** Department of Obstetrics, Obstetrics and Gynecology Hospital of Fudan University, Shanghai, China

**Keywords:** pre-pregnancy body mass index, hypertension disorder in pregnancy, gestational diabetes mellitus, macrosomia, preterm birth, low birthweight

## Abstract

**Objective:**

Adverse pregnancy outcomes have been related to obesity and thinness; however, the changing trends of the specific outcome with pre-pregnancy BMI remain unknown. The aim of this study was to investigate the change in risk trends of specific adverse outcomes for different pre-pregnancy BMI and analyze the recommended BMI range for pre-pregnancy counseling.

**Methods:**

Data were extracted from the medical records of 39 public hospitals across 14 provinces in China from 2011 to 2012. The eligibility criteria were singleton birth with delivery week ≥28 weeks. Join-point analysis was adopted to explore changing trends with pre-pregnancy BMI and calculate slopes and join points of different pregnancy complications.

**Results:**

A total of 65,188 women were eligible for analysis. There were three categories of trend style. Continuously increasing trends were linear for intrahepatic cholestasis of pregnancy, postpartum hemorrhage, and low 1-min Apgar score, and non-linear for cesarean delivery with one join point of BMI 23, hypertension disorder in pregnancy with two join points of BMI 20 and 28, gestational diabetes mellitus with one join point of BMI 22, and macrosomia with one join point of BMI 19. The trend was continuously and linearly decreasing for anemia. The bidirectional trends were downward and upward for premature rupture of the membrane with join BMI 22, preterm premature rupture of the membrane with join BMI 22, placenta abruption with join BMI 23, preterm birth with join BMI 19, and low birth weight with join BMI 19.

**Conclusions:**

The changes in the trends of specific outcomes differed with pre-pregnancy BMI. Our results suggested that a pre-pregnancy BMI ranging between 19 and 23 may help reduce the risk of poor maternal and neonatal outcomes.

## Introduction

Numerous studies have reported that abnormal pre-pregnancy body mass index (BMI) could be associated with adverse maternal or neonatal outcomes. Existing evidence shows that women with overweight or obesity before pregnancy are at increased risk for gestational diabetes mellitus (GDM), hypertensive disorders in pregnancy (HDP), macrosomia, cesarean section, and neonatal mortality (1–5). In contrast, low BMI before pregnancy may contribute to a higher risk of preterm birth, fetal growth restriction (FGR), and small for gestational age (SGA) status (6).

Most previous studies examined maternal and neonatal outcomes across different BMI groups based on the Institute of Medicine (IOM) classification. However, the influence of pre-pregnancy BMI on pregnancy outcomes may be accumulated from quantitative change to qualitative change, and the risk of adverse outcomes may accelerate after a certain point, which has not been previously reported. Besides, the pattern of effect of pre-pregnancy BMI on morbidity is diverse for each complication. Therefore, the objective of the current study was to investigate the change in risk trends of specific adverse outcomes for different pre-pregnancy BMI and to analyze the recommended BMI range for women before pregnancy.

## Materials and Methods

**Figure 1:**
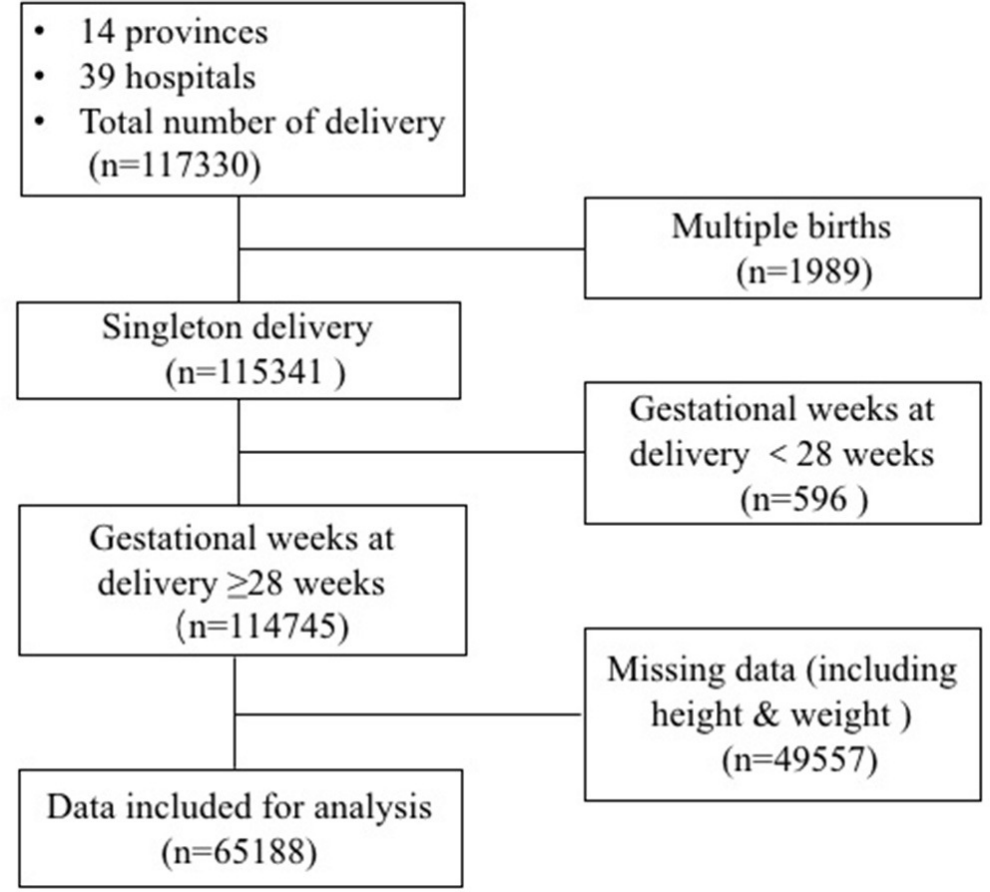
Patient flow chart.

Data were extracted from the medical records of 39 public hospitals across 14 provinces in China between 2011 and 2012. The inclusion criteria encompassed women with singleton pregnancies and gestational week of birth ≥28 weeks. A total of 65,188 women were included in the final analysis (Figure 1).

All women were required to provide their medical records within the first 12 weeks of gestation and accept systematic antenatal care until delivery. Relevant data were collected from these medical records by trained hospital staff. Women's height was measured, and their pre-pregnancy weight was self-reported during the first antenatal visit. Gestational week of birth and pregnancy outcomes were extracted from the discharge records after delivery. The birthweight of the newborn was obtained within 1 h after delivery. BMI was calculated as weight (kg)/height^2^ (m^2^).

Adverse maternal outcomes included cesarean section, HDP, GDM, anemia, intrahepatic cholestasis of pregnancy (ICP), premature rupture of membrane (PROM), preterm premature rupture of membrane (PPROM), postpartum hemorrhage, and placental abruption. Adverse neonatal outcomes included preterm birth (28–37 weeks), macrosomia (>4,000 g), low birthweight (<2,500 g), and low 1-min Apgar score (≤7). Other adverse outcomes like birth defects or stillbirth were not analyzed for low morbidity. Diagnoses were recorded as International Classification of Diseases-10 codes by healthcare providers in the hospital. Women with pre-pregnancy BMI of 15 or lower as well as women with pre-pregnancy BMI of 34 or higher were combined into one separate group because of the small number of such cases. BMI-specific rates for each pregnancy outcome indicator were calculated. The Hospital Committee for Medical Research Ethics approved the study under the ethic approval code 2017–18. All of the methods used in the study were in accordance with the relevant guidelines, and informed consent to participate was obtained from all subjects.

SPSS 21 was used for preparation and descriptive analyses of all data. Baseline characteristics were presented as numbers (percentage) for categorical variables. Join-point regression analysis was used to test whether an apparent change in temporal trend is statistically significant, where several linear segments are connected together at the “join points.” It was applied in the analysis of cancer temporal trends previously and developed to be applied in other fields. In our study, join-point regression analysis (version 4.5.0.1) was adopted to establish the trend of pregnancy outcomes with maternal pre-pregnancy BMI. The crude rates of adverse pregnancy outcomes were used as dependent variables, and maternal pre-pregnancy BMI was used as the independent variable. Poisson variance was used to estimate the non-constant variance of segmental models by assuming that the dependent variable counts follow a Poisson distribution. The join-point analysis identified the best fitting piecewise continuous linear model by Bayesian information criterion. This approach allowed us to identify the specific BMI when there were significant changes in the trend and to estimate the magnitude of the increase or the decrease in each segment by estimating the slopes.

## Results

A total of 65,188 women were eligible for analysis. The baseline data of all enrolled women in different pre-pregnancy BMI groups are shown in Table 1. Results showed that there were no differences in demographic characteristics between the four groups.

**Table 1:** Demographics of women in different pre-pregnancy BMI groups.

**Characteristic**	**Underweight** **(7,840)**	**Normal** **(49,214)**	**Overweight** **(7,131)**	**Obese** **(1,003)**	***P*** **-value**
**Maternal age**					0.06
≤34	702 0 (89.97%)	44,103 (89.91%)	6,304 (88.83%)	875 (87.94%)	
35~39	630 (8.07%)	3,995 (8.14%)	641 (9.03%)	95 (9.55%)	
≥40	153 (1.96%)	952 (1.94%)	152 (2.14%)	25 (2.51%)	
**Parity**					0.10
Primipara	6,564 (83.72%)	40,732 (82.77%)	5,877 (82.41%)	842 (83.95%)	
Multipara	1,276 (16.28%)	8,482 (17.23%)	1,254 (17.59%)	161 (16.05%)	
**Maternal educational level**					0.51
High (universities and above)	3,676 (47.68%)	23,053 (47.81%)	3,390 (47.82%)	477 (48.77%)	
Middle (secondary schools)	2,445 (31.72%)	15,035 (31.18%)	2,275 (32.09%)	301 (30.78%)	
Low (primary schools and lower)	1,588 (20.60%)	10,134 (21.02%)	1,424 (20.09%)	200 (20.45%)	
**Maternal self-reported smoking history**					0.36
Yes	34 (0.43%)	190 (0.39%)	21 (0.29%)	6 (0.60%)	
No	7,806 (99.57%)	49,024 (99.61%)	7,110 (99.71%)	997 (99.40%)	
**Maternal self-reported drinking history**					0.37
Yes	139 (1.77%)	656 (1.33%)	86 (1.21%)	13 (1.30%)	
No	7,701 (98.23%)	48,558 (98.67%)	7,045 (98.79%)	990 (98.70%)	

*BMI, body mass index*.

Adverse maternal and neonatal outcomes are shown in Table 2. A total of <5% of data for adverse outcomes was missing. Maternal complications included cesarean delivery in 53.76% (35046), HDP in 5.14% (3350), GDM in 4.71% (3072), anemia in 5.77% (3760), ICP in 0.54% (353), PROM in 15.60% (10169), PPROM in 2.05% (1335), placenta abruption in 0.49% (321), and postpartum hemorrhage in 3.61% (2355) women. Adverse neonatal outcomes included preterm birth in 4.94% (4571), macrosomia in 7.01% (4571), low birth weight in 4.94% (3223), and a low 1-minute Apgar score (≤7) in 2.35% (1532) women. Besides, result showed that there were differences between groups of different pre-pregnancy BMI in the aspects of cesarean section, HDP, GDM, anemia, ICP, PPROM, postpartum hemorrhage, preterm birth, macrosomia and low birthweight (*P* < 0.05).

**Table 2:** Incidence of adverse maternal and fetal outcomes of women in different pre-pregnancy BMI group.

**Adverse outcomes**	**All women** **(65,188)**	**Underweight** **(7,840)**	**Normal** **(49,214)**	**Overweight** **(7,131)**	**Obese** **(1,003)**	***P*** **-value**
**Maternal complications**						
Cesarean section	35,046 (53.76%)	3,403 (43.41%)	26,397 (53.64%)	4,526 (63.47%)	720 (71.78%)	<0.01
HDP	3,350 (5.14%)	227 (2.90%)	2,188 (4.45%)	721 (10.11%)	214 (21.34%)	<0.01
GDM	3,072 (4.71%)	205 (2.61%)	1,974 (4.01%)	704 (9.87%)	189 (18.84%)	<0.01
Anemia	3,760 (5.77%)	446 (5.69%)	2,869 (5.83%)	394 (5.53%)	51 (5.08%)	0.56
ICP	353 (0.54%)	35 (0.45%)	267 (0.54%)	39 (0.55%)	12 (1.20%)	0.03
PROM	10,169 (15.60%)	1,300 (16.58%)	7,590 (15.42%)	1,115 (15.64%)	164 (16.35%)	0.06
PPROM	1,335 (2.05%)	201 (2.56%)	921 (1.87%)	181 (2.54%)	32 (3.19%)	<0.01
Postpartum hemorrhage	2,355 (3.61%)	240 (3.06%)	1,713 (3.48%)	348 (4.88%)	54 (5.38%)	<0.01
Placental abruption	321 (0.49%)	43 (0.55%)	239 (0.49%)	35 (0.49%)	4 (0.40%)	0.87
**Adverse neonatal outcomes**						
Preterm birth	3,768 (5.78%)	494 (6.30%)	2,679 (5.44%)	507 (7.11%)	88 (8.77%)	<0.01
Macrosomia	4,571 (7.01%)	299 (3.84%)	3,227 (6.59%)	883 (12.45%)	162 (16.25%)	<0.01
Low birthweight	3,223 (4.94%)	496 (6.38%)	2,298 (4.69%)	368 (5.19%)	61 (6.12%)	<0.01
Low 1-min Apgar score (≤7)	1,532 (2.35%)	177 (2.28%)	1,134 (2.32%)	191 (2.70%)	30 (3.01%)	0.12

*HDP, Hypertension disorder in pregnancy;GDM:gestational diabetes mellitus; ICP, intrahepatic cholestasis of pregnancy; PROM: premature rupture of membrane; PPROM, preterm premature rupture of membrane*.

**Figure 2:**
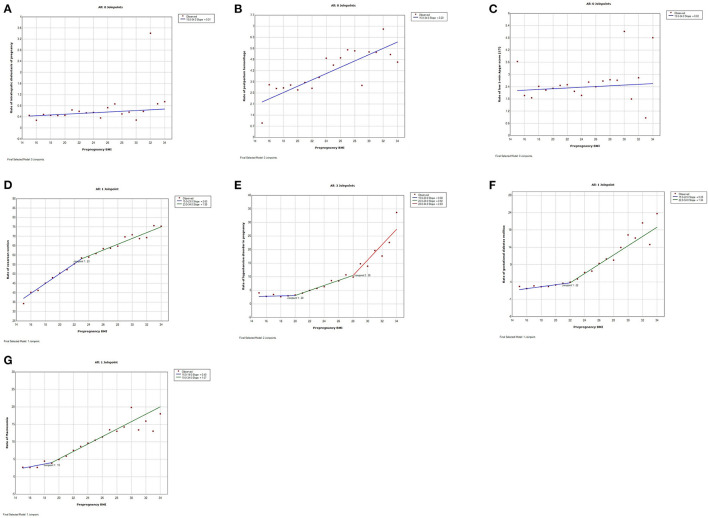
Trends of adverse pregnancy outcomes in relation to maternal pre-pregnancy BMI by join-point analysis (increasing trends). **(A–C)** Continuous linear increase for intrahepatic cholestasis of pregnancy (ICP), postpartum hemorrhage, and low 1-min Apgar score (≤7). **(D–G)** Continuous non-linear increase for cesarean section, hypertension disorder in pregnancy (HDP), gestational diabetes mellitus (GDM), and macrosomia.

**Figure 3:**
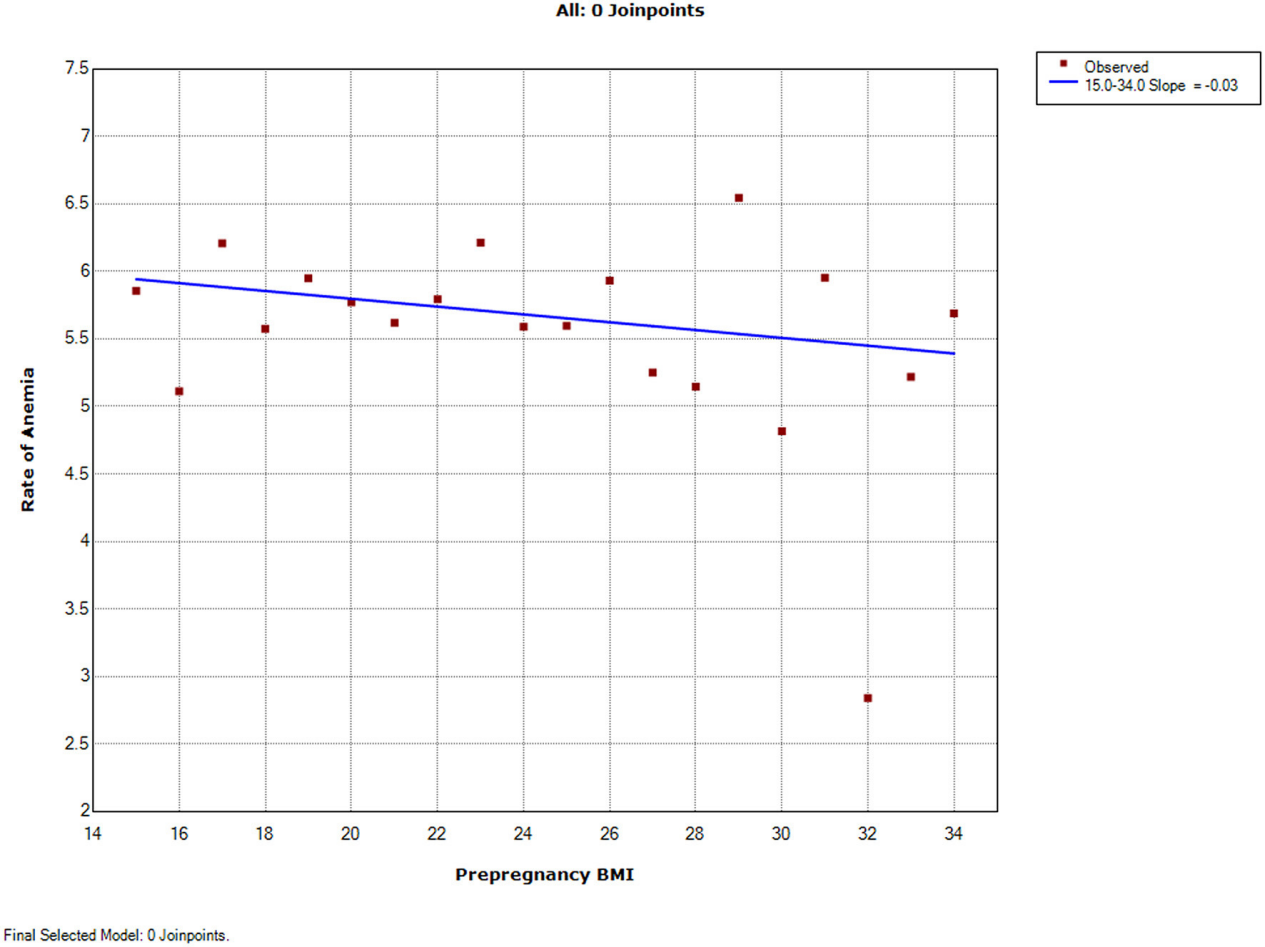
Trends of adverse pregnancy outcomes with maternal pre-pregnancy BMI by join-point analysis (decreasing trends). Continuous linear decrease for anemia.

**Figure 4:**
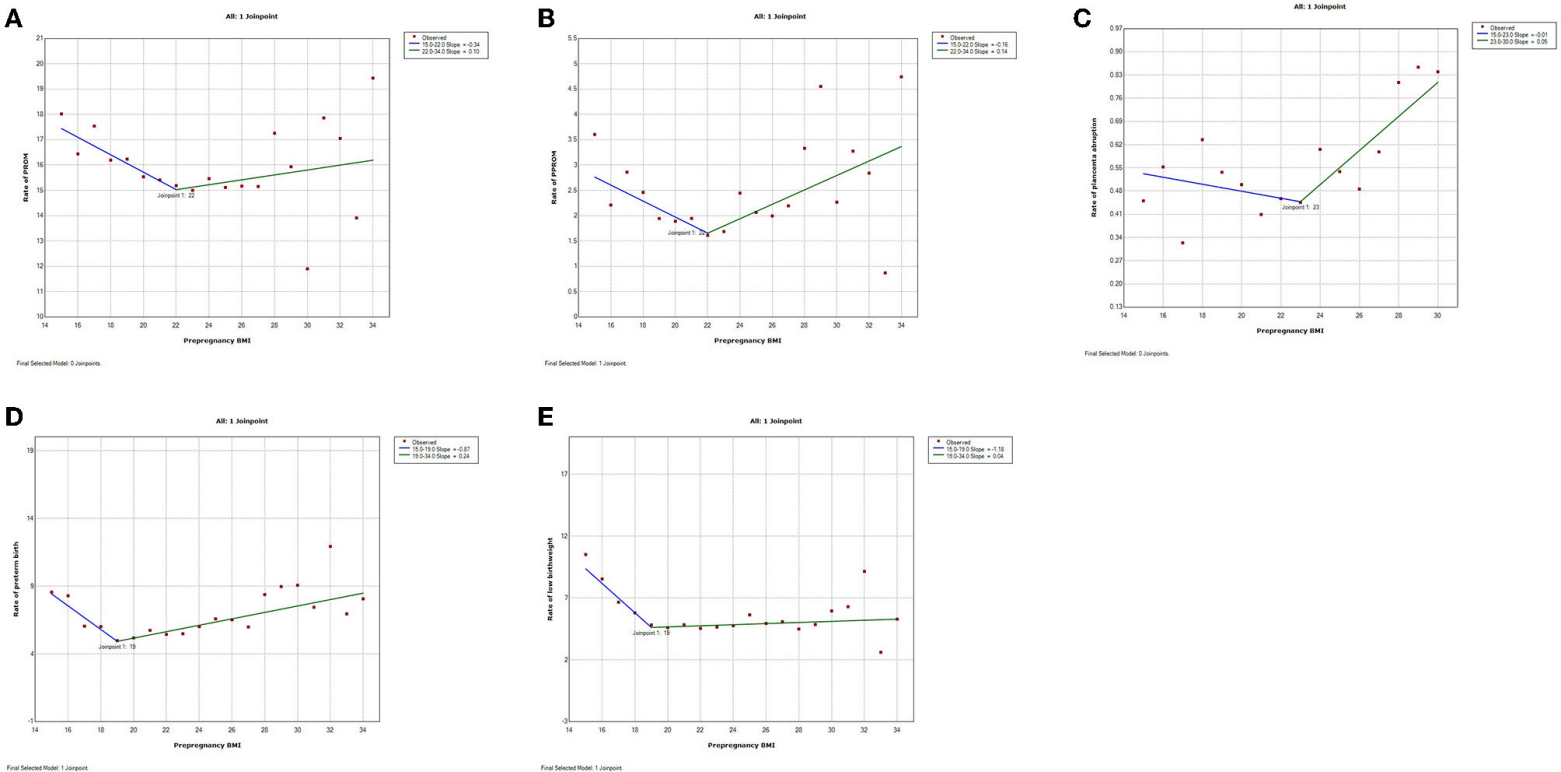
Trends of adverse pregnancy outcomes with maternal pre-pregnancy BMI by join-point analysis (Bidirectional trends). **(A–E)** Bidirectional changes with down-and-upward trends for premature rupture of membrane (PROM), preterm premature rupture of membrane (PPROM), placenta abruption, preterm birth, and low birthweight.

The trends of adverse pregnancy outcomes with maternal pre-pregnancy BMI assessed by join-point analysis are shown in Table 3. Seven adverse outcomes revealed a continuously increasing trend with maternal pre-pregnancy BMI, one had a continuously decreasing trend, and five had a bidirectional trend, with a decreasing trend in thin women and an increasing trend in obese women at different nadir BMI (Figures 2–4).

**Table 3:** Join-point analysis of changing trend of adverse pregnancy outcomes with pre-pregnancy BMI.

**Complications**	**Trend 1**			**Trend 2**			**Trend 3**		
	**BMI**	**Slope**	***P*** **-value**	**BMI**	**Slope**	***P*** **-value**	**BMI**	**Slope**	***P*** **-value**
Cesarean section	15–23	2.63	<0.01^*^	23–34	1.55	<0.01^*^			
HDP	15–20	0.08	0.68	20–28	0.92	<0.01^*^	28–34	2.83	<0.01^*^
GDM	15–22	0.28	<0.01^*^	22–34	1.34	<0.01^*^			
Anemia	15–34	−0.03	0.27						
ICP	15–34	0.01	0.16						
PROM	15–22	−0.34	0.02^*^	22–34	0.10	0.33			
PPROM	15–22	−0.16	0.08	22–34	0.14	0.03^*^			
Postpartum hemorrhage	15–34	0.20	<0.01^*^						
Placenta abruption	15–23	−0.01	0.51	23–34	0.05	0.10			
Preterm birth	15–19	−0.87	0.05	19–34	0.24	<0.01^*^			
Low birthweight	15–19	−1.18	<0.01^*^	19–34	0.04	0.23			
Macrosomia	15–19	0.40	0.21	19–34	1.07	<0.01^*^			
Low 1-min Apgar score (≤7)	15–34	0.02	0.45						

*HDP, Hypertension disorder in pregnancy;GDM:gestational diabetes mellitus; ICP, intrahepatic cholestasis of pregnancy; PROM: premature rupture of membrane; PPROM: preterm premature rupture of membrane*.

The trends were continuously increasing for seven adverse pregnancy outcomes (Figure 1). The trend of ICP, postpartum hemorrhage, and low 1-min Apgar score increased linearly with pre-pregnancy BMI ranging from 15 to 34 without join-points (slope 0.01, 0.20, and 0.02%, respectively). The trends of the other four adverse pregnancy outcomes were continuous but non-linear with different join-points. The trend of cesarean delivery increased rapidly from BMI 15 to 23 (slope 2.63%) and then a little slowly from BMI 23 to 34 (slope 1.55%). The trend of HDP increased a little from BMI 15 to 20 (slope 0.08%), and then slightly increased from BMI 20 to 28 (slope 0.92%), followed by a significant increase from BMI 28 to 34 (slope 2.83%). The trend of GDM slightly increased from BMI 15 to 22 (slope 0.28%) and then increased rapidly from BMI 22 to 34 (slope 1.34%). The trend of macrosomia slightly increased from BMI 15 to 19 (slope 0.40%), while a rapid increase ensued from BMI 19 to 34 (slope 1.07%). The trend for anemia was continuously and linearly decreasing (slope -0.03%) (Figure 2).

Trends were bidirectional, downward-to-upward, with different nadir BMI for PROM, PPROM, placenta abruption, preterm birth, and low birthweight (Figure 3). The trend of PROM was decreasing before BMI 22 and increasing after that (slope−0.34% before BMI 22 and slope 0.10% after BMI 22). Similarly, the trend of PPROM was decreasing before BMI 22 and increasing after that (slope−0.16% before BMI 22 and slope 0.14% after BMI 22). The trend of placenta abruption was downward (slope−0.01%) before BMI 23 and upward (slope 0.05%) after it. The trend of low birthweight was downward before BMI 19 (slope−1.18%) and upward after it (slope 0.04%).

## Discussion

Our data revealed the trend changes of pre-pregnancy BMI in relation to different adverse outcomes. Seven adverse pregnancy outcomes including cesarean section, HDP, GDM, ICP, postparturm hemorrhage, macrosomia, and low 1-min Apgar score revealed a continuously increasing trend with maternal pre-pregnancy BMI; anemia had a continuously decreasing trend, while five other adverse outcomes, including PROM, PPROM, placenta abruption, preterm birth, and low birth weight had a bidirectional trend that was decreasing in thin women and increasing in obese women at different nadir BMI.

In most previous studies, adverse pregnancy outcomes related to different pre-pregnancy BMI were mostly analyzed across different BMI groups. In our study, join-point regression was adopted to investigate the continuous changing trend of adverse pregnancy outcomes in relation to pre-pregnancy BMI. As a result, the trend of risky pregnancy started to change at join BMI, meaning the impact of risk factors changed with pre-pregnancy BMI, even though the change could be insignificant. Join point is the theoretical point at which two adjacent trends cross. Our join BMI was mostly at the range of normal BMI of the IOM classification, but it was more specific, and the results were in line with those of previous studies on the effect of maternal pre-pregnancy BMI (analyzed by BMI groups) (1, 6, 7). Therefore, there is good reason to believe that most of our results on the association between abnormal pre-pregnancy BMI and adverse pregnancy outcomes are applicable to other reports.

Our results revealed a continuously increasing trend of HDP, GDM, ICP, macrosomia, cesarean section, postpartum hemorrhage, and low 1-min Apgar score, suggesting that only high pre-pregnancy BMI was a risk factor for these outcomes. The impact of high pre-pregnancy BMI on these outcomes was similar to previous reports (8–19). According to existing studies, obese (BMI 30 to 33.9) and morbidly obese (BMI > 40) primigravid women have 3 and 7 times higher risks of pre-eclampsia, respectively (8). Our study showed the incidence of HDP increased when pre-pregnancy BMI reached 20, which is traditionally considered normal BMI, and significantly increased when pre-pregnancy BMI reached 28. Similarly, the incidence slope of GDM increased when pre-pregnancy BMI reached 22 and significantly increased when pre-pregnancy BMI reached 27. While other studies have found similar results, they made no recommendations regarding BMI (13, 14). A previous study showed that compared with the normal group, the obese group was at 1.7 times higher risk of macrosomia, while the risk in the overweight group did not increase (15). Our results revealed that pre-pregnancy BMI affected the incidence of macrosomia in a continuous and linear way. Our results were also consistent with earlier reports, which have shown an association between increasing BMI and cesarean delivery and postpartum hemorrhage (16). This may be subsequent to induction, including altered uterine contractility combined with dysfunctional labor (17, 18). Besides, the link between high pre-pregnancy BMI and low 1 min Apgar score may be secondary to the result of increasing pregnancy complications. On the contrary, the negative association between low BMI and maternal anemia may be due to poor nutrition, including iron, folic acid, and other micronutrient deficiencies (19).

Low and high pre-pregnancy BMI affected some adverse outcomes, including PROM, PPROM, placenta abruption, preterm birth, and low birth weight. Previous studies showed that low maternal BMI was associated with more spontaneous preterm deliveries and low birth weight (20). Nevertheless, our results showed that trends of preterm birth, as well as low birth weight, were bidirectional, with a decrease in BMI lower than 19 and an increase higher than 19, suggesting that mainly being underweight (usually defined as BMI < 18.5) was a risk factor for preterm birth and low birthweight. Among other populations, including those with normal weight, overweight and obese, the risk slightly went up as BMI increased, which may be due to other increasing pregnancy complications. Similar trends were also found in PROM and placenta abruption with join BMI of 22 and 23, respectively, which have not been previously reported in other researches.

The strengths of this study included large, retrospective, and continuous data of pregnant women collected from 39 hospitals across 14 provinces in a multi-center and cross-sectional way, including rural and urban populations, thus making the sample quite representative. The large sample size also made it possible to calculate the rate of different pre-pregnancy BMI and describe the incidence trend with BMI by join-point regression analysis. While most of the previous studies had grouped comparison design according to IOM guidelines, join-point analysis added priority to continuously investigate BMI.

However, there are some limitations in the present study that should be considered. First, pre-pregnancy BMI was determined by self-reported weight at their first antenatal visit, and there may be a possibility of confounding bias given the retrospective study design. Second, factors including gravity and parity, occupation, education, and unhealthy habits like smoking or drinking were not adjusted, which may cause bias. Large-scale prospective studies may be needed to investigate these problems further.

## Conclusion

Maternal HDP, GDM, ICP, macrosomia, cesarean section, postpartum hemorrhage, and low 1-min Apgar score were only affected by high pre-pregnancy BMI, whereas maternal anemia was only affected by low pre-pregnancy BMI. Low and high pre-pregnancy BMI affected the risk of PROM, PPROM, placenta abruption, preterm birth, and low birth weight in different modes, and the satisfactory BMI before pregnancy appeared to be between 19 and 23. The estimated pre-pregnancy BMI could be helpful in identifying targeted BMI and providing pre-pregnancy counseling for reducing the risk of poor maternal and neonatal outcomes. Besides, according to our results, clinicians could particularly pay attention to specific pregnancy complications of high risks during pregnancy for women with different pre-pregnancy BMI.

## Data Availability Statement

The raw data supporting the conclusions of this article will be made available by the authors, without undue reservation.

## Ethics Statement

The Hospital Committee for Medical Research Ethics approved the study. The patients/participants provided their written informed consent to participate in this study.

## Author Contributions

RH contributed to writing of the manuscript. HY contributed to data collection and data analysis. XL contributed to the planning of the project and revising of the manuscript. All authors have read and approved the manuscript.

## Funding

This study was supported by the National Natural Science Foundation of China (grant no. 81571460).

## Conflict of Interest

The authors declare that the research was conducted in the absence of any commercial or financial relationships that could be construed as a potential conflict of interest.

## Publisher's Note

All claims expressed in this article are solely those of the authors and do not necessarily represent those of their affiliated organizations, or those of the publisher, the editors and the reviewers. Any product that may be evaluated in this article, or claim that may be made by its manufacturer, is not guaranteed or endorsed by the publisher.

## Abbreviations

BMI: body mass index
HDP: hypertension disorder in pregnancy
GDM: gestational diabetes mellitus
ICP: intrahepatic cholestasis of pregnancy
PROM: premature rupture of membrane
PPROM: preterm premature rupture of membrane
FGR: Fetal growth restriction
SGA: Small for gestational age.
